# The Brine Bath Treatment of Cholera

**Published:** 1892-09-10

**Authors:** 


### The Brine Bath Treatment of Cholera.

In this year of grace, 1892, when our much boasted measures against the importation and spread of infectious diseases are being sorely tried, and when the natural prophylactics of our sea-begirt island are being set at defiance by the microbe of cholera, it may, perhaps, interest the inhabitants of this same island to learn that salt water is alleged to act, not only as a natural antiseptic and prophylactic against the bacillus of cholera, but that it has also most remarkable curative properties when employed as an external application.

In the year 1832, when England was first visited by a severe epidemic of Asiatic cholera, the peculiar efficacy of salt water as an external application to patients suffering from this disease was first clearly demonstrated by the late Sir Charles Hastings. The circumstances of the discovery are as follows:—

A village in the neighbourhood of Worcester, famous since the time of the Romans for its salt works, was attacked by an outbreak of cholera so suddenly and severely that a panic seized upon the inhabitants, and so great was the alarm that the victims found but scant attention at the hands of medical men, nurses, or relatives. A temporary hospital was improvised out of a disused salt work, and, to continue in the words of the late Doctor Roden: "Still the difficulty remained of procuring anyone to take charge of it. At length a man and his wife were found willing to undertake the management, the man's duties being, upon notice given, to fetch the patient from wherever he might be and bring him to the hospital, and prepare a hot bath, and if it was a man to bath him and place him in bed, and further, when a fatal result occurred, to convey the body to the cemetery. . . . On one occasion, a patient being brought in during the night, it was found that there was no hot water to be had for a bath. Under these circumstances the man fetched buckets of boiling brine out of a neighbouring salt work in order to prepare one speedily. The effect of this novel bath upon the patient was to resuscitate him; the skin became warm, voice and pulse returned, and the patient rapidly recovered. The magic result amazed even the medical staff, and henceforward during the epidemic the hot brine bath was used to every case, and with the most favourable result."

That such effects may well follow the treatment of cholera by brine baths is not at variance with scientific experience, or contrary to the principles of rational treatment. Death from cholera is due to exhaustion, probably from the dissemination throughout the system of some alkaloid or leucomaïne, the product of bacterial activity. To combat this exhaustion, the interests of the patient are best subserved by preserving him from all useless expenditure of energy. By immersing a patient in a brine bath at the normal temperature of the body, the general temperature is soon brought within normal limits, and, inasmuch as the specific gravity of the brine closely approximates to that of the body, any exertion from the muscular exercise of placing the body in the position of greatest ease is minimised. And when placed in a brine bath at such a temperature the patient finds himself exposed to an environment best adapted for the physical requirements of the case, whilst floating on a water-bed of the most perfect description, he is in comparative ease and comfort. Besides, the soothing effect, the mitigation of exhausting evacuations, the relief of cramp in the legs are benefits which cannot be exaggerated.

It is highly improbable that there is any peculiar or subtle virtue in the water of Droitwich, or any other similar bathing resort; the virtue lies in its cheapness and the ease with which large supplies of water can be obtained. Concentrated sea water, or water brought up to the necessary specific gravity by the addition of common salt, would doubtless effect the same purpose.

This treatment, the efficacy of which has been proved beyond a doubt in past epidemics, certainly deserves another trial should cholera break out in districts where brine can easily be acquired.

